# Traditional Human Populations and Nonhuman Primates Show Parallel Gut Microbiome Adaptations to Analogous Ecological Conditions

**DOI:** 10.1128/mSystems.00815-20

**Published:** 2020-12-22

**Authors:** Ashok K. Sharma, Klara Petrzelkova, Barbora Pafco, Carolyn A. Jost Robinson, Terence Fuh, Brenda A. Wilson, Rebecca M. Stumpf, Manolito G. Torralba, Ran Blekhman, Bryan White, Karen E. Nelson, Steven R. Leigh, Andres Gomez

**Affiliations:** a Department of Animal Science, University of Minnesota, St. Paul, Minnesota, USA; b Institute of Vertebrate Biology, Czech Academy of Sciences, Brno, Czech Republic; c Institute of Parasitology, Biology Centre, Czech Academy of Sciences, Ceske Budejovice, Brno, Czech Republic; d Department of Anthropology, University of North Carolina, Wilmington, Wilmington, North Carolina, USA; e WWF‐CAR, Bangui, Central African Republic; f Carl Woese Institute of Genomic Biology, University of Illinois, Urbana, Illinois, USA; g Department of Microbiology, University of Illinois, Urbana, Illinois, USA; h Department of Anthropology, University of Illinois, Urbana, Illinois, USA; i J. Craig Venter Institute, La Jolla, California, USA; j Department of Genetics, Cell Biology, and Development, University of Minnesota, Minneapolis, Minnesota, USA; k Department of Animal Science, University of Illinois, Urbana, Illinois, USA; l Department of Anthropology, University of Colorado, Boulder, Colorado, USA; University of Connecticut

**Keywords:** gut microbiome, metagenomics, gorillas, traditional agriculturalists, hunter-gatherers

## Abstract

Compared with urban-industrial populations, small-scale human communities worldwide share a significant number of gut microbiome traits with nonhuman primates. This overlap is thought to be driven by analogous dietary triggers; however, the ecological and functional bases of this similarity are not fully understood. To start addressing this issue, fecal metagenomes of BaAka hunter-gatherers and traditional Bantu agriculturalists from the Central African Republic were profiled and compared with those of a sympatric western lowland gorilla group (Gorilla *gorilla gorilla*) across two seasons of variable dietary intake. Results show that gorilla gut microbiomes shared similar functional traits with each human group, depending on seasonal dietary behavior. Specifically, parallel microbiome traits were observed between hunter-gatherers and gorillas when the latter consumed more structural polysaccharides during dry seasons, while small-scale agriculturalist and gorilla microbiomes showed significant functional overlap when gorillas consumed more seasonal ripe fruit during wet seasons. Notably, dominance of microbial transporters, transduction systems, and gut xenobiotic metabolism was observed in association with traditional agriculture and energy-dense diets in gorillas at the expense of a functional microbiome repertoire capable of metabolizing more complex polysaccharides. Differential abundance of bacterial taxa that typically distinguish traditional from industrialized human populations (e.g., *Prevotella* spp.) was also recapitulated in the human and gorilla groups studied, possibly reflecting the degree of polysaccharide complexity included in each group’s dietary niche. These results show conserved functional gut microbiome adaptations to analogous diets in small-scale human populations and nonhuman primates, highlighting the role of plant dietary polysaccharides and diverse environmental exposures in this convergence.

**IMPORTANCE** The results of this study highlight parallel gut microbiome traits in human and nonhuman primates, depending on subsistence strategy. Although these similarities have been reported before, the functional and ecological bases of this convergence are not fully understood. Here, we show that this parallelism is, in part, likely modulated by the complexity of plant carbohydrates consumed and by exposures to diverse xenobiotics of natural and artificial origin. Furthermore, we discuss how divergence from these parallel microbiome traits is typically associated with adverse health outcomes in human populations living under culturally westernized subsistence patterns. This is important information as we trace the specific dietary and environmental triggers associated with the loss and gain of microbial functions as humans adapt to various dietary niches.

## Abstract (precis)

The results of this study highlight parallel gut microbiome traits in human and nonhuman primates, depending on subsistence strategy. Although these similarities have been reported before, the functional and ecological bases of this convergence are not fully understood.

## INTRODUCTION

The gut microbiome of different primate species has been shown to be phylogenetically conserved and inherited in a vertical manner (1, 2). This phylogenetic signal is maintained, even when individual primate hosts face natural dietary shifts, likely reflecting constraints imposed by host physiological evolution (3). However, similarities in gut microbiome traits between closely and distantly related primates can still be observed, likely due to shared ecological niches, and independent from geographical location or host genetic similarity (4, 5). Analyzing the functional basis of similar microbiome traits between different primates, including humans, could shed light on the ecological forces that have impacted the human microbiome in the context of subsistence gradients and health and disease phenotypes. For instance, while the gut microbiome of populations that rely on westernized subsistence strategies has adapted rapidly to industrialized dietary behaviors and lifestyles (6–8), traditional human populations worldwide (hunter-gatherers and small-scale agriculturalists) share numerous compositional and functional traits with nonhuman primates (4, 5, 9). These observations may indicate that the specific ecological and subsistence forces shaping the gut microbiome of traditional human populations may be analogous to those seen in nonhuman primates. In contrast, adaptations to industrialized subsistence and lifestyle patterns have triggered the loss of those microbiome features shared between nonhuman primates and human populations (7), which has been hypothesized to adversely impact the physiological landscape of human populations living in a culturally westernized context (9–11).

To shed light on the ecological basis of similar and divergent microbiome traits between humans and nonhuman primates, we investigated functional microbiome adaptations to different subsistence strategies in humans and measured the extent to which these adaptations align with those seen in a closely related nonhuman primate across analogous subsistence gradients. In this regard, we have previously documented significant compositional microbiome distinctions between the gut microbiomes of sympatric hunter-gatherers and small-scale agriculturalists (7), likely driven by adaptations to process energy-dense, more processed diets by the latter (12). In addition, we have shown that when western lowland gorillas transition from dry to wet seasons, their gut microbiome composition corresponds with gut metabolome traits associated with increased energetic turnover (13). As such, we expect parallel functional changes between gorillas and traditional populations in response to analogous ecological conditions. Here, we hypothesized that functional microbiome adaptations that distinguish foraging from agricultural subsistence in humans (7) are analogous to those seen in wild western lowland gorillas (Gorilla *gorilla gorilla*) when shifting feeding behaviors between foliage/leaf-based diets and high-energy, ripe fruit consumption during dry and rainy seasons, respectively. We discuss our findings in the context of microbiome adaptations to subsistence shifts in humans and nonhuman primates and the factors that shape the human microbiome as we know it today.

## RESULTS

### Characteristics of the metagenome data set.

After collecting fecal samples of BaAka hunter-gatherers (*n* = 14), Bantu agricultural populations (*n* = 14), and sympatric wild western lowland gorillas (G. gorilla *gorilla*) across dry (*n* = 11) and wet seasons (*n* = 12) in the Dzanga Sangha Protected Areas (Central African Republic), we profiled gut microbiome functions via shotgun metagenomic sequencing. We obtained a total of 979,507,504 host-filtered shotgun reads (average of 19,206,030 reads per sample; range, 5,701,216 to 33,876,024) (see Data Set S1, tab 1, in the supplemental material). The shotgun metagenomic reads were mapped (average mapping rate of ∼81.38% [Data Set S1, tab 1]) onto a total nonredundant gene set (a total of 4,298,551 open reading frames), constructed by combining all predicted genes (a total of 12,260,992) from assembled contigs in each sample, followed by clustering. Overall, gorillas, regardless of season, exhibited higher functional diversity compared with the two human groups; however, Bantu agriculturalists always exhibited the lowest gene content richness and diversity (Kruskal-Wallis test; *P* < 0.05) (see Fig. S1a and b in the supplemental material). To mine for functional gut microbiome distinctions between BaAka and Bantu and gorillas across dry and wet seasons, a total of 3,804,658 filtered genes (present in at least three samples) were quantified and mapped against KEGG, carbohydrate-active enzymes (CAZy), and xenobiotic degradation enzymes (XDEs) databases (see Materials and Methods). From the KEGG pathway analysis, a total of 242,349 KEGG genes (6.4% of filtered gene set), 5,747 KEGG modules (KEGG orthologs [KOs]), 1391 EC numbers, and 330 pathways were quantified in all samples.

10.1128/mSystems.00815-20.1 FIG S1 Gene diversity in the gut microbiome of western lowland gorillas across seasons of variable dietary intake and in humans under two different subsistence strategies. (a) Total number of observed genes (b) Shannon’s H index. Kruskal-Wallis test was used for significance testing. The center values indicate the medians, and error bars depict the SD. ns, not significant; *, *P* < 0.05. Download FIG S1, TIF file, 1.4 MB. Copyright © 2020 Sharma et al. 2020 Sharma et al. This content is distributed under the terms of the Creative Commons Attribution 4.0 International license.

10.1128/mSystems.00815-20.10 DATA SET S1 Supplemental data sets of each comparison are provided in separate tabs of Data Set S1. Download Data Set S1, XLSX file, 2.1 MB. Copyright © 2020 Sharma et al. 2020 Sharma et al. This content is distributed under the terms of the Creative Commons Attribution 4.0 International license.

### Functional microbiome profiles in hunter-gatherers, agriculturalists, and gorillas across wet and dry seasons.

**FIG 1:**
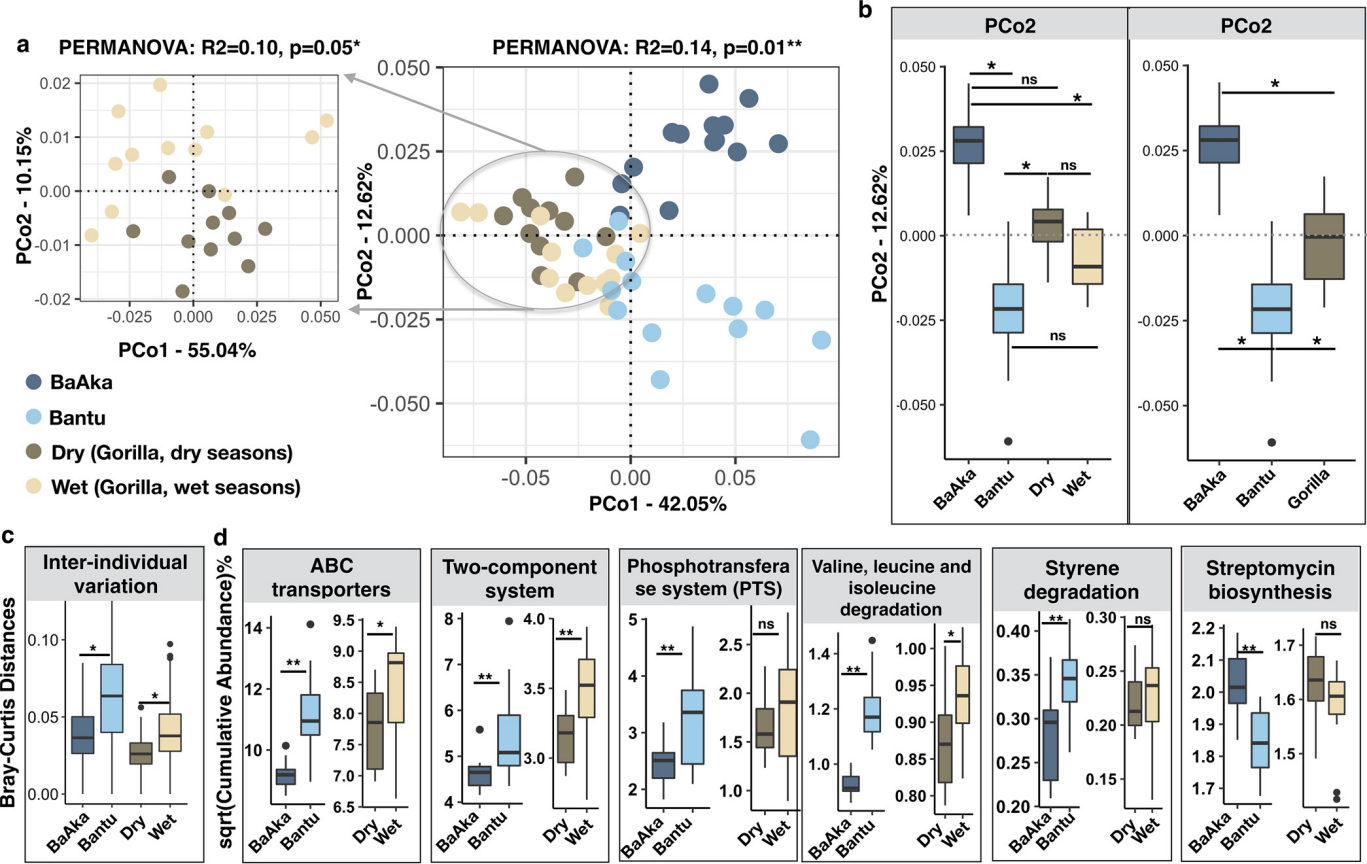
Functional adaptations in the gut microbiome of western lowland gorillas across seasons of variable dietary intake and in humans under two different subsistence strategies. (a) Principal-coordinate analysis using Bray-Curtis distances generated from the relative abundances of KEGG pathways shows functional distinctions between the microbiome of gorillas across dry (dark wheat) and wet (light wheat) seasons and between BaAka hunter-gatherers and Bantu agriculturalists (PERMANOVA: *R*^2^ = 0.14, *P* = 0.01**). The amplified ordination panel on the left specifically shows functional distinctions between gorillas across seasons (PERMANOVA: *R*^2^ = 0.10, *P* = 0.05*). (b) Ordination scores along PCo2 reflect functional similarities between BaAka hunter-gatherers and gorillas in dry seasons and between Bantu agriculturalists and gorillas in wet seasons. (c) Higher interindividual variations were observed in Bantu agriculturalists and gorillas during the wet season. (d) Relative abundances of significantly discriminating pathways identified using gene set enrichment analysis further highlights the similarity between Bantu and gorillas during the wet season and BaAka and gorillas during the dry season. The square root (sqrt) of cumulative abundance is shown on the *y* axes. The color key in panel a applies to all panels. A nonparametric two-sided Wilcoxon rank sum test was used for testing the box plot distributions. The center values indicate the median values, and error bars depict the standard deviations (SD). ns, not significant; *, *P* < 0.05; **, *P* < 0.01.

Analyses of KEGG pathways (relative abundances) showed clear distinctions among the fecal metagenomes of the BaAka, Bantu, and the gorillas across wet and dry seasons (Bray-Curtis distance, principal-coordinate analysis [PCoA], permutational multivariate analysis of variance [PERMANOVA], *R*^2^ = 0.14, *P* = 0.001) (Fig. 1a). However, inspection of PCoA ordination scores indicated that, compared with Bantu agriculturalists, BaAka hunter-gatherer metagenomes shared more functional features with those of western lowland gorillas, particularly, during the dry season, when gorillas consumed more leaves, herbs, and fibrous fruits. In contrast, the functional gut microbiome landscape of Bantu agriculturalists shared more common traits with gorillas during the wet season, when they consume more ripe fruit (Fig. 1b).

To address factors driving this interspecies similarity, the abundance distribution of specific KEGG pathways was analyzed. For instance, the BaAka exhibited significantly less interindividual variability in KEGG pathways compared to the Bantu agriculturalists. This trait was also observed in gorillas during the dry season, in contrast to when they consumed more ripe, digestible fruit sources during the rainy season (Fig. 1c). In general, more discriminant KOs were detected characterizing the Bantu agriculturalists (144 versus 27 in the BaAka) and the wet season in gorillas (695 versus 526 in the dry season) (Data Set S1, tabs 2 and 3). However, upon further inspection of all detected pathways (Fig. S2a and b and Data Set S1, tabs 4 and 5), some interspecies commonalities were found; for example, pathways involved in membrane transport (ABC transporters, phosphotransferase system), regulatory signal transduction systems (two-component system), amino acid catabolism (valine, leucine, and isoleucine) and xenobiotic degradation (styrene) were consistently more abundant in Bantu agriculturalists and gorillas during the wet season. These pathways were significantly depleted in the BaAka and gorillas during the dry season; the BaAka and gorillas exhibited an increased abundance of microbial genes involved in streptomycin biosynthesis compared with the Bantu and gorillas during the wet season (Fig. 1d).

10.1128/mSystems.00815-20.2 FIG S2 Significantly discriminating pathways identified using reporter feature analysis. Differences in the gut microbiome of BaAka hunter-gatherers and Bantu agriculturalists (a) and gorillas during dry and wet seasons (b). Positive scores of Stat (dist.dir.up) on the *x* axis showing significant enriched pathways in BaAka hunter-gatherers and gorillas during dry season, whereas negative scores of Stat (dist.dir.up) on the *x* axis show significantly enriched pathways in Bantu agriculturalists and gorillas during wet seasons. Download FIG S2, TIF file, 2.6 MB. Copyright © 2020 Sharma et al. 2020 Sharma et al. This content is distributed under the terms of the Creative Commons Attribution 4.0 International license.

Additionally, lipid metabolic activities in the gut microbiome of these communities were analyzed. PCoA analysis on relative abundances of pathways involved in lipid metabolism showed minor differences between the fecal metagenomes of the BaAka, Bantu, and the gorillas across wet and dry seasons (PERMANOVA, *R*^2^ = 0.13, *P* = 0.004). However, closer inspection shows functional lipid metabolic differences between the microbiomes of BaAka hunter-gatherers and Bantu agriculturalists (PERMANOVA, *R*^2^ = 0.17, *P* = 0.01), while no significant differences were found between gorillas across seasons of variable dietary intake (PERMANOVA, *R*^2^ = 0.08, *P* = 0.09) (Fig. S3a). Specifically, among all lipid metabolic pathways analyzed, synthesis and degradation of ketone bodies were consistently abundant in Bantu agriculturalists and gorillas during the wet season (Fig. S3b). Of note, metabolism of linoleic acid was highly conserved in the BaAka and gorillas during wet seasons (Fig. S3b). This observation could be related to higher consumption of linolenic acid-rich diets, such as those found in oils of vegetable origin from nuts, seeds, and fruits. These foods, which are consumed more by the gorillas in the wet seasons and by the BaAka (12, 14), have been associated with optimal cardiovascular health (15, 16).

10.1128/mSystems.00815-20.3 FIG S3 Lipid metabolism in the gut microbiome of gorillas across two seasons of variable dietary intake and in humans under two different subsistence strategies. (a) Principal-coordinate analysis using Bray-Curtis distances generated from the relative abundances of pathways involved in lipid metabolism shows distinctions between the microbiome BaAka hunter-gatherers and Bantu agriculturalists (PERMANOVA: *R*^2^ = 0.17, *P* = 0.01**), and no distinctions between gorillas across dry (dark wheat) and wet (light wheat) seasons (PERMANOVA: *R*^2^  = 0.08, *P* = 0.09). (b) Relative abundances of significantly discriminating lipid metabolic pathways are shown in the boxplot. The color key in panel a applies to all panels. A nonparametric two-sided Wilcoxon rank sum test was used for testing the box plot distributions. The center values indicate the medians, and error bars depict the SD. ns, not significant; *, *P* < 0.05; **, *P* < 0.01. Download FIG S3, TIF file, 2.7 MB. Copyright © 2020 Sharma et al. 2020 Sharma et al. This content is distributed under the terms of the Creative Commons Attribution 4.0 International license.

**FIG 2:**
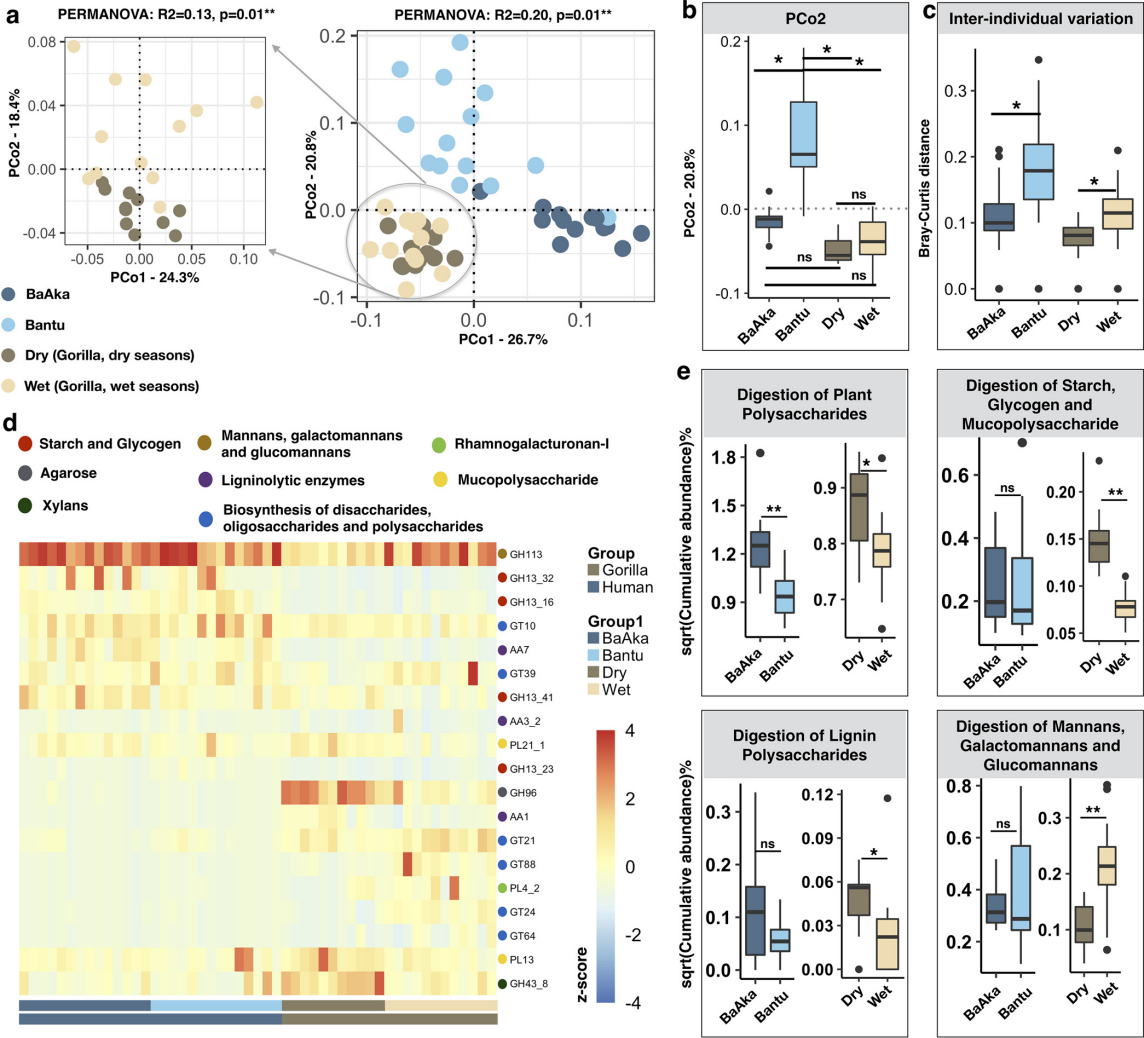
Carbohydrate-degrading capabilities in the gut microbiome of gorillas across two seasons of variable dietary intake and in humans under two different subsistence strategies. (a) Principal-coordinate analysis using Bray-Curtis distances generated from the CAZyome shows distinctions in carbohydrate-degrading capabilities in the microbiome of gorillas across dry and wet seasons and between BaAka hunter-gatherers and Bantu agriculturalists (PERMANOVA: *R*^2^ = 0.20, *P* = 0.01**). The amplified ordination panel on the left specifically shows functional distinctions between gorillas across seasons (PERMANOVA: *R*^2^ = 0.13, *P* = 0.01**). (b) PCo2 ordination score reflects functional similarities of BaAka hunter-gatherers with gorillas in both dry and wet seasons. (c) Higher interindividual variability in CAZyme content was observed in Bantu agriculturalists and gorillas during the wet season. (d) Heatmap of CAZy families and subfamilies shows discriminant patterns among groups and subgroups. Two-sided Wilcoxon rank sum test was applied for each pair (dry versus wet season and BaAka versus Bantu) (false-discovery rate [FDR]-corrected *P* < 0.05). Color code of each CAZyme shows the broad carbohydrate utilization capabilities. The heatmap is color coded based on the row normalized z-scores. (e) Distinctions in broad CAZyme categories among human and gorilla groups and subgroups, plotted by their respective distributions. The color key in panel a applies to all panels. A nonparametric two-sided Wilcoxon rank sum test was used for testing the box plot distributions. The center values indicate the medians, and error bars depict the SD. ns, not significant; *, *P* < 0.05; **, *P* < 0.01.

To further investigate the specific dietary factors driving this interspecies functional similarity, an analysis of carbohydrate-active enzymes (CAZymes) was conducted. A total of 19,783 (0.52% of filtered gene set) CAZy genes, belonging to 362 CAZy families, were identified in the complete metagenomic pool, indicating a distinct CAZyme repertoire in the gut microbiome of BaAka, Bantu, and gorillas across seasons (Bray-Curtis, PCoA, PERMANOVA, *R*^2^ = 0.20, *P* = 0.01) (Fig. 2a). However, as observed with KEGG pathways, the CAZyome (collection of all CAZyme-coding genes) distinctions between the Bantu and BaAka were also analogous to those seen in gorillas shifting from fruit- to leaf-based diets, with the CAZyome of the hunter-gatherers showing more similarities with that of gorillas regardless of season (Fig. 2b). As with KEGG pathways, one of the shared traits detected reflected higher interindividual variation in the CAZyome of Bantu agriculturalists and gorillas during the wet season (Fig. 2c). Also, although the abundance of main CAZy classes was similar between humans and gorillas, showing predominance of glycoside hydrolases (GH) and transferases (GT) in both primate species (Fig. S4a), discriminant and similar patterns in the abundance of several CAZy families between the BaAka and Bantu (Fig. S5a and Data Set S1, tab 6) and dry and wet seasons in gorillas were detected (Fig. S5b and Data Set S1, tab 7). For example, GH subfamily 113, involved in the metabolism of mannans, galactomannans, and glucomannans was more abundant in both human groups and in gorillas during the wet season, while GH subfamilies involved in the metabolism of starch and glycogen (GH13) were more abundant in humans. CAZy subfamilies involved in the metabolism of xylans (GH43), mucopolysaccharides (polysaccharide lyase PL13), and agarose (GH96) were more abundant in gorillas (Fig. 2d).

10.1128/mSystems.00815-20.4 FIG S4 Carbohydrate- and lipid-degrading capabilities in the gut microbiome of gorillas across two seasons of variable dietary intake and in humans under two different subsistence strategies. (a) The line plot shows a similar distribution of CAZyme classes in all groups, with glycoside hydrolases (GHs) being the prevalent class in both species. (b) Distinctions in broad CAZyme categories among human and gorilla groups and subgroups, plotted by their respective distributions. Association of genes involved in the synthesis and degradation of ketone bodies with total gene diversity of CAZymes (c) and cumulative abundances of total CAZymes (d). Download FIG S4, TIF file, 2.8 MB. Copyright © 2020 Sharma et al. 2020 Sharma et al. This content is distributed under the terms of the Creative Commons Attribution 4.0 International license.

10.1128/mSystems.00815-20.5 FIG S5 Significantly discriminating CAZymes across different groups. (a) Microbiome in the guts of humans under two different subsistence strategies. Positive and negative fold changes reflect higher enrichment in the BaAka hunter-gatherers and Bantu agriculturalists, respectively. (b) In the gut microbiome of western lowland gorillas across seasons. Positive and negative fold changes reflect higher enrichment in gorillas during dry and wet seasons, respectively. Significantly discriminating CAZymes belong to glycoside hydrolases (GH13, GH27, GH96, GH43, and GH113), glycosyl transferases (GT24, GT88, GT39, GT64, GT10, and GT21), polysaccharide lyases (PL4, PL21, and PL13), auxiliary activities (AA1, AA7, and AA3), and carbohydrate-binding modules (CBM73, CBM65, CBM46, CBM43, and CBM40). These discriminating CAZymes are shown in magenta. Download FIG S5, JPG file, 0.2 MB. Copyright © 2020 Sharma et al. 2020 Sharma et al. This content is distributed under the terms of the Creative Commons Attribution 4.0 International license.

When CAZy families were divided into specific classes based on carbohydrate utilization capabilities (Data Set S1, tab 8), their cumulative proportions showed some interspecies similarities based on subsistence strategy (Fig. 2e). Primarily, CAZymes involved in the digestion of plant polysaccharides were enriched in gorillas during the dry season and in BaAka hunter-gatherers. For other broad categories, clear differences were found only in gorillas with CAZymes involved in digestion of starch, glycogen, mucopolysaccharides, lignin, and algal polysaccharides distinguishing the wild apes during the dry season. In contrast, digestion of mannans, galactomannans, and glucomannans and biosynthesis of polysaccharides seemed to be more prevalent when gorillas consumed more fruit during rainier periods of the year. Although similar interspecies patterns were observed, no significant differences were detected in these broad categories between BaAka hunter-gatherers and Bantu agriculturalists (Fig. 2e and Fig. S4b). However, we found negative associations between synthesis and degradation of ketone bodies, an important pathway involved in lipid metabolism, and the abundance and diversity of carbohydrate-active enzymes (CAZymes) (Spearman *r*^2^ = −0.53 and *r*^2^ = −0.69, respectively, Fig. S4c and d).

**FIG 3:**
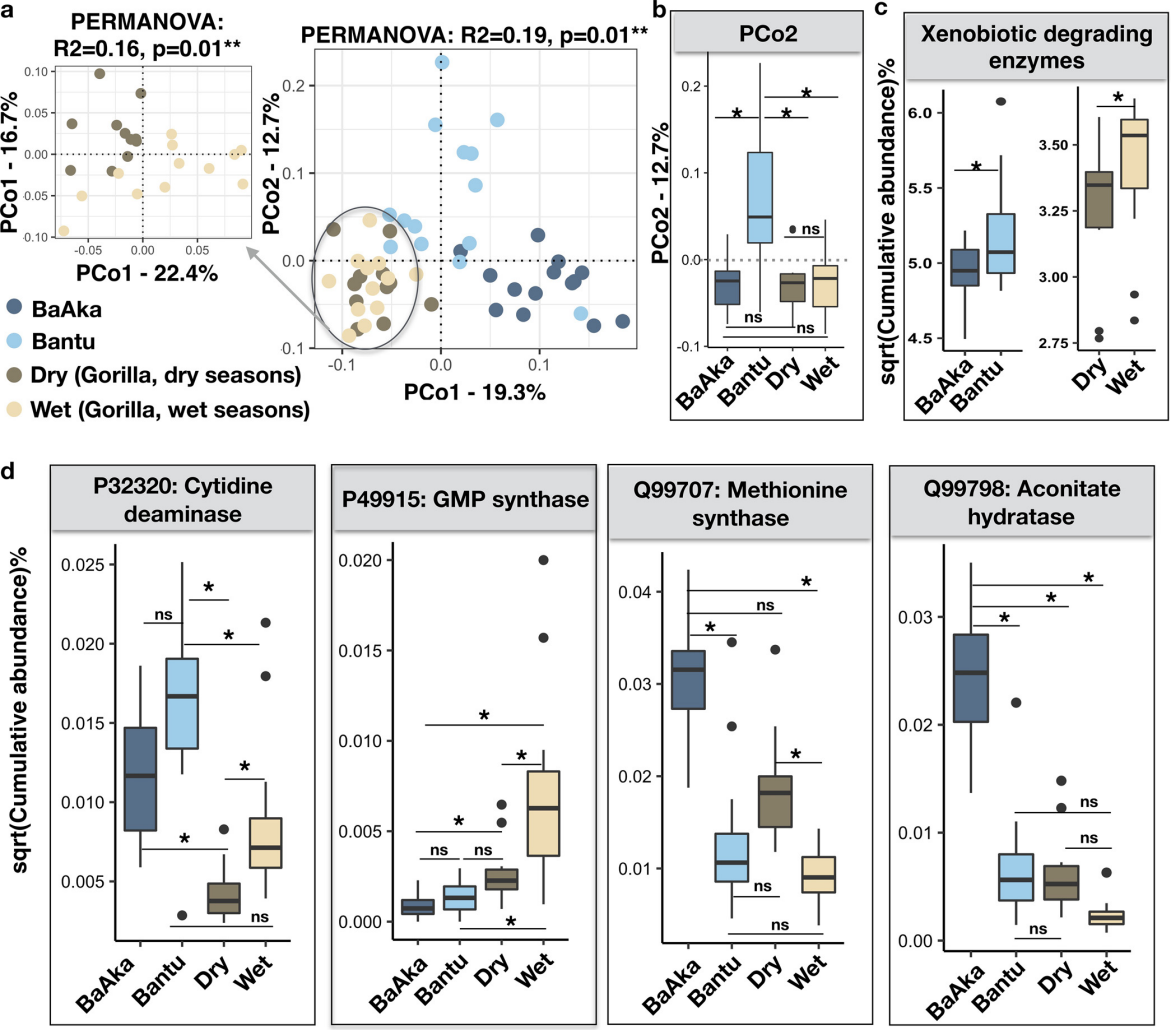
Xenobiotic-degrading capabilities in the gut microbiome of gorillas across two seasons of variable dietary intake and in humans under two different subsistence strategies. (a) Principal-coordinate analysis using Bray-Curtis distances generated from the relative abundances of XDEs shows distinctions in xenobiotic-degrading capabilities in the microbiome of gorillas across dry and wet seasons and between BaAka hunter-gatherers and Bantu agriculturalists (PERMANOVA: *R*^2^ = 0.19, *P* = 0.01**). The amplified ordination panel on the left specifically shows functional distinctions between gorillas across seasons (PERMANOVA: *R*^2^ = 0.16, *P* = 0.01**). (b) PCo2 ordination score reflects functional similarities of BaAka hunter-gatherers with gorillas in both dry and wet seasons. (c) The distribution of XDEs across population shows higher abundance in Bantu agriculturalists and gorillas during the wet season. (d) Relative abundances of selected significantly discriminating XDEs are plotted by their respective distributions. The color key in panel a applies to all panels. A nonparametric two-sided Wilcoxon rank sum test was used for testing the box plot distributions. The center values indicate the medians, and error bars depict the SD. ns, not significant; *, *P* < 0.05; **, *P* < 0.01.

To assess other dietary or environmental exposure drivers of the interspecies similarities observed, functional microbiome adaptations to xenobiotics were also analyzed. We identified a total of 95 xenobiotic degradation enzymes (XDEs), indicating inter- and intraspecies distinctions and similarities (Bray-Curtis, PCoA, PERMANOVA, *R*^2^ = 0.19, *P* = 0.01), and highlighting similarities in XDE content between the hunter-gatherers and the gorillas (Fig. 3a and b). Although both human groups exhibited higher content of XDEs, the agriculturalists and gorillas during the wet season exhibited the greatest abundance (Wilcoxon rank sum test; *P* = 0.04 for BaAka hunter-gatherers versus Bantu agriculturalists, and *P* = 0.05 for dry versus wet season) (Fig. 3c). In general, more discriminant XDEs (odds ratio > 2) characterized the Bantu (26 versus 8 in the BaAka) and the gorillas during the wet season (12 versus 8 in the dry season) (Fig. S6a and b and Data Set S1, tabs 9 and 10). However, some interspecies commonalities were found; for instance, the abundance of cytidine deaminase (NCBI:protein accession no. P32320) was higher in the Bantu agriculturalists, and significantly higher in gorillas during the wet season. In contrast, abundance of methionine synthase (Q99707) was higher in the BaAka and tended to be higher when gorillas consumed more leaves (Fig. 3d). Abundance of aconitate hydratase (Q99798) was also higher in the BaAka, compared to any other group, while wet season gorilla microbiomes showed greater abundances of GMP synthase (P49915) (Fig. 3d).

10.1128/mSystems.00815-20.6 FIG S6 Significantly discriminating xenobiotic-degrading enzymes. Differences in the gut microbiome of BaAka hunter-gatherers and Bantu agriculturalists (a) and gorillas during dry and wet seasons (b). Positive fold changes reflect higher enrichment in the gut microbiome of BaAka hunter-gatherers and gorillas during the dry season, whereas negative fold changes reflect higher enrichment in the gut microbiome of Bantu agriculturalists and gorillas during wet seasons. Download FIG S6, TIF file, 2.3 MB. Copyright © 2020 Sharma et al. 2020 Sharma et al. This content is distributed under the terms of the Creative Commons Attribution 4.0 International license.

### Associations between functional and taxonomic microbiome profiles.

**FIG 4:**
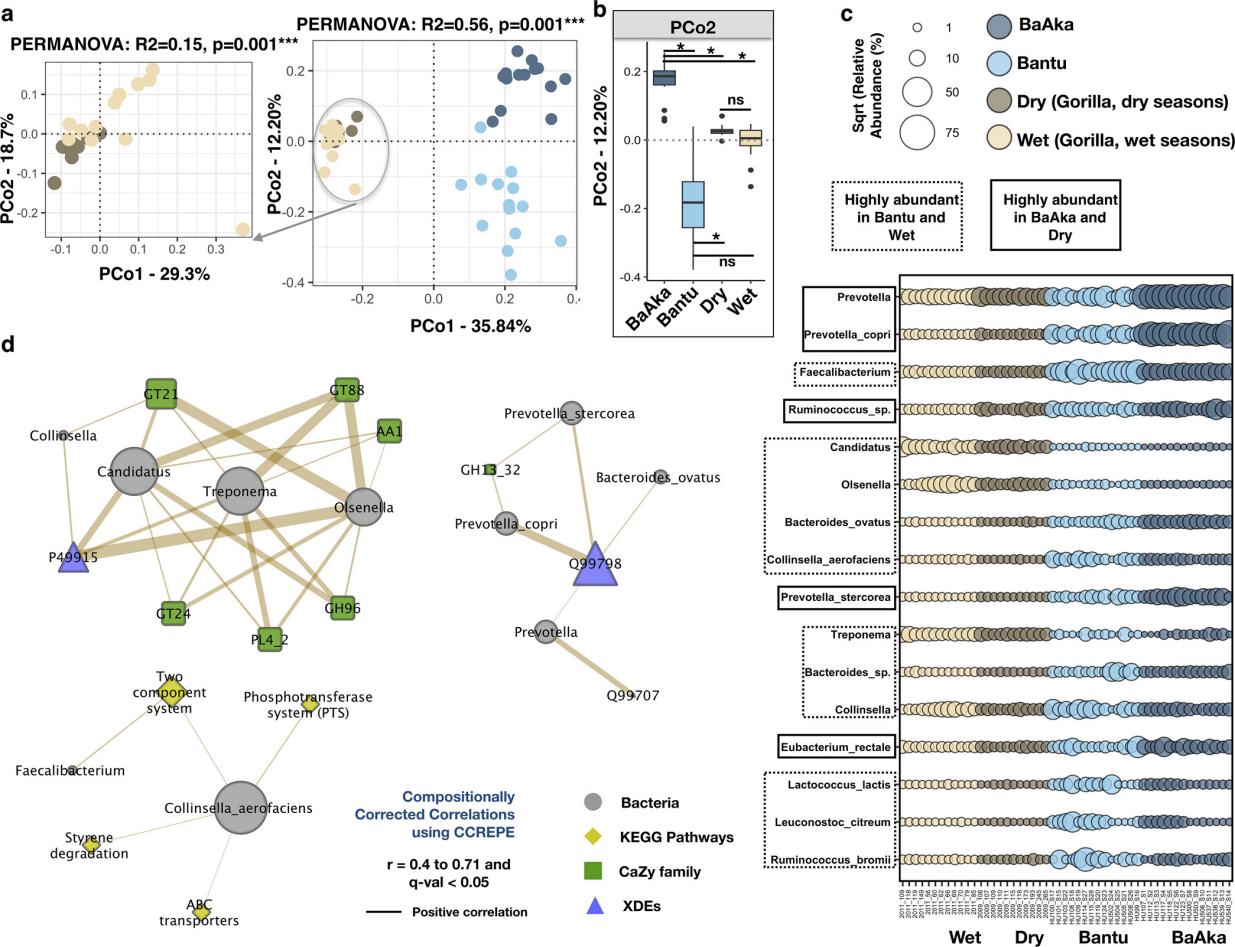
Taxonomic abundances in the gut microbiome of gorilla across two seasons of variable dietary intake and of humans under two different subsistence strategies. Taxonomic assignments obtained from shotgun metagenomic data were used for this analysis. (a) Principal-coordinate analysis using Bray-Curtis distances generated from the relative abundances of bacterial taxa (NCBI plus HMP) shows distinctions in the microbial composition of gorillas across dry and wet seasons and between BaAka hunter-gatherers and Bantu agriculturalists (PERMANOVA: *R*^2^ = 0.56, *P* = 0.001***) and gorillas during dry versus wet seasons separately (left graph, PERMANOVA: *R*^2^ = 0.15, *P* = 0.001***). The amplified ordination panel on the left specifically shows distinctions in the microbial composition between gorillas across seasons (PERMANOVA: *R*^2^ = 0.15, *P* = 0.01**). (b) Ordination scores along PCo2 did not show similarity in BaAka hunter-gatherers or Bantu agriculturalists with gorillas in dry or wet seasons. (c) Bubble plot of discriminating taxa selected using indicator species analysis (total mean relative abundance > 1%, indval > 0.4 and *P* < 0.05). (d) Correlation network analysis between significantly discriminating bacterial taxa and functional profiles. The plot was constructed in CytoScape using positive compositionally corrected correlations (0.4 < *r* < 0.71 and *q* value < 0.05) calculated using CCREPE. Symbols and colors represent different microbial nodes, whereas edge patterns represent the strength and direction of correlation. The color key at the top of panel c applies to all panels. ns, not significant; *, *P* < 0.05; **, *P* < 0.01.

We identified a total of 810 bacterial species in the sampled gut metagenomes. Analyses on species distribution show significant discrimination among all four groups (Bray-Curtis, PCoA, PERMANOVA, *R*^2^ = 0.56, *P* = 0.001) (Fig. 4a) but also some interspecies commonalities, with hunter-gatherers showing more taxonomic similarities with gorillas regardless of season (Fig. 4b). This analysis also showed five taxa simultaneously enriched in the BaAka hunter-gathers and in gorillas during the dry season: *Prevotella* spp., Prevotella copri, *Ruminococcus* spp., Prevotella stercorea, and Eubacterium rectale. In contrast, 11 taxa were enriched in the Bantu agriculturalists and in gorillas when more fruit was consumed: *Faecalibacterium*, *Candidatus*, *Olsenella*, Bacteroides ovatus, Collinsella aerofaciens, *Treponema*, *Bacteroides* spp., *Collinsella*, Lactococcus lactis, Leuconostoc citreum, and Ruminococcus bromii (Fig. 4c and Data Set S1, tabs 11 and 12).

To infer the functional potential of these taxa, a co-occurrence analysis with the functions previously detected as similar or discriminant was conducted and depicted in three subnetworks (SNs) (compositionally corrected correlations 0.4 < *r* < 0.7, *q* value < 0.05). For example, in SN1, unidentified *Olsenella* found as markers of the Bantu and wet season microbiomes in gorillas coabounded with several CAZymes; glycosyl transferases (GH) subfamilies 21, 88, and 24, polysaccharide lyases (PL 4_2), multicopper oxidases (auxiliary activity, AA1), and GH96. This cluster also showed association with XDE P49915 (GMP synthase). In SN2, Collinsella aerofaciens, which showed the greatest abundance in the agriculturalists and the gorillas during the wet season, showed coabundance patterns with genes involved in styrene degradation, phosphotransferase system (PTS) systems, ABC transporters, and two-component systems, with *Faecalibacterium* also coabundanding with the latter. Prevotella copri and *Prevotella stercorea*, markers of the microbiomes of hunter-gatherers and gorillas during the dry season, coabounded with markers of starch and glycogen degradation (GH13) and XDE Q99798 (aconitate hydratase). Abundances of Bacteroides ovatus and an unknown *Prevotella* were also associated with both XDEs Q99798 and Q99707 (methionine synthase) (Fig. 4d and Data Set S1, tab 13).

### Carbohydrate utilization capabilities of shared taxonomic traits between humans and gorillas.

**FIG 5:**
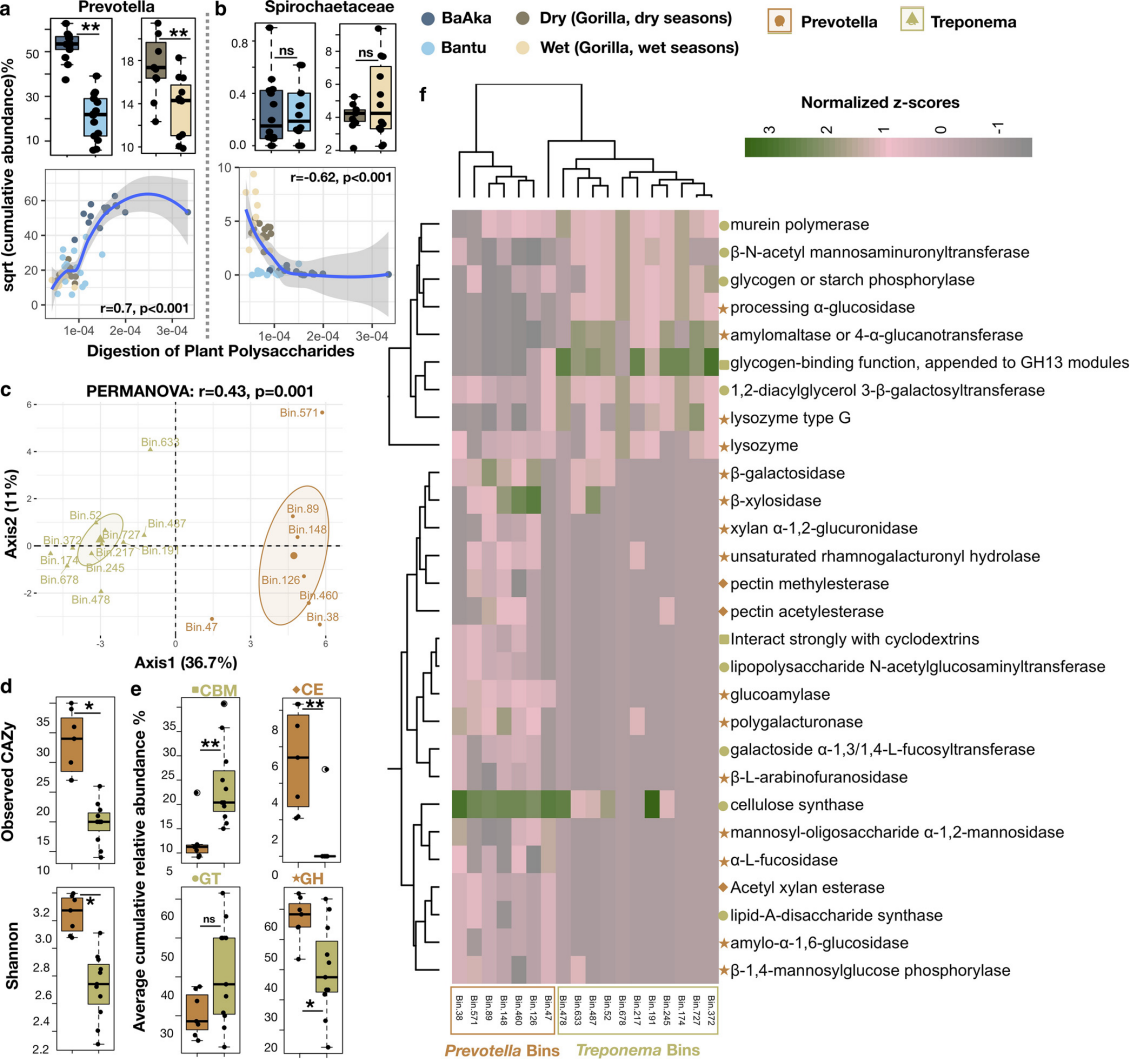
Functional and genome reconstruction analyses of *Prevotella* and *Spirochaetaceae* in the gut microbiome of gorillas across two seasons of variable dietary intake and in humans under two different subsistence strategies. From the taxonomic analysis, cumulative abundance of all discriminating taxa belonging to *Prevotella* (a) and *Spirochaetaceae* (b) were used to assess their distribution and association with the digestion of plant polysaccharides. (c) Principal-component analysis generated from the relative abundances of CAZy families in *Prevotella* and *Treponema* bins (PERMANOVA: *R*^2^ = 0.43, *P* = 0.001***). (d) Higher diversity of carbohydrate-active enzymes was observed in *Prevotella* compared to *Treponema* bins. (e) Relative abundances of broad CAZy classes found in these bins were plotted by their respective distributions. (f) Heatmap of significantly discriminating CAZy families showing differences between *Prevotella* and *Treponema* bins (two-sided Wilcoxon rank sum test for each pair, dry versus wet and BaAka hunter-gatherers versus Bantu agriculturalists, *P* < 0.05, Data Set S1, tab 17). Color code represents bins, whereas symbols represent CAZy classes as shown in panel e. Heatmap is color coded based on normalized z-scores. A nonparametric two-sided Wilcoxon rank sum test was used for testing the box plot distributions. The center values indicate the medians, and error bars depict the SD. ns, not significant; *, *P* < 0.05; **, *P* < 0.01.

Given the associations detected between specific marker taxa and functional profiles and the importance of *Prevotella* and *Treponema* as taxa that consistently differentiate nonhuman primates and traditional human populations from industrialized societies (5, 17, 18), we sought to further investigate their degrading capabilities and possible dietary associations. The cumulative abundance of all discriminating taxa belonging to the genus *Prevotella* coincided with the patterns mentioned above; that is, this taxon was more abundant in the BaAka and gorillas during the dry season. *Spirochaetaceae* were always more abundant in both gorilla groups compared to humans, but this taxon did not follow differential trends across subsistence gradients in humans or gorillas (Fig. 5a and b). These analyses also revealed corresponding associations between abundance of the genus *Prevotella* and all CAZymes involved in the degradation of plant polysaccharides. Conversely, this association was negative for *Spirochaetaceae* (Fig. 5a and b). Representative genomes of these two taxa were reconstructed, with a total of 1,388 genomic bins recovered from the 51 metagenomes. The completeness and contamination of each bin were estimated using the presence or absence of lineage-specific marker genes. Of these, 335 bins (average completeness = 78.33, average contamination = 1.46, average number of scaffolds = 187.23) from dry and wet season gorilla samples (Fig. S7a, left panel, and Data Set S1, tab 14) and 257 bins (average completeness = 72.62, average contamination = 1.48, average number of scaffolds = 234.85) from BaAka hunter-gatherers and Bantu agriculturalists (Fig. S7b, left panel, and Data Set S1, tab 15) were recovered. A total of seven genomic bins were assigned to *Prevotella* and 11 to the *Spirochaetaceae* family in all four groups (detailed statistics provided in Fig. S7a and b and Data Set S1, tab 16). There were no direct bins assigned to the *Treponema* genome; hence, *Spirochaetaceae* bins were considered for further analysis.

10.1128/mSystems.00815-20.7 FIG S7 Assessment of the quality of reconstructed genomic bins. Estimated completeness versus contamination and completeness versus number of scaffolds of genomic bins recovered from the metagenomic data from gorilla populations in the left panel (a) and from the human population in the right panel (b). Only genomic bins with >50% completeness were retained. Near-complete genomes (completeness ≥ 90%; contamination ≤ 5%) are shown in green, medium-complete genomes (completeness ≥ 70%; contamination ≤ 10%) are shown in turkish green, and partial genomes (completeness ≥ 50%; contamination ≤ 4%) are shown in in dark gray. Quality assessment of genomic bins belonging to *Prevotella* genus (in blue) and *Spirochaetaceae* family (in orange) from gorilla population in the left panel and from the human population in the right panel. Download FIG S7, TIF file, 2.8 MB. Copyright © 2020 Sharma et al. 2020 Sharma et al. This content is distributed under the terms of the Creative Commons Attribution 4.0 International license.

First, we examined the distribution and sequence similarity of single-copy marker genes of the selected bins against *Prevotella* and *Treponema* reference genomes. This procedure led to the identification of Prevotella copri DSM 18205 and Treponema succinifaciens DSM 2489 as the most similar species to the bins recovered, as reported previously for other traditional populations worldwide (18, 19) (Fig. S8a and b). A principal-component analysis based on the relative abundance of CAZy families in each constructed bin was performed, showing substantial differences in the carbohydrate utilization capabilities between the reconstructed genomes of *Prevotella* and *Treponema* (PERMANOVA, *R*^2^ = 0.43, *P* = 0.001, Fig. 5c). For example, *Prevotella* bins showed higher abundance and diversity of total CAZyme repertoires (Fig. 5d) and higher abundance of carbohydrate esterases (CE) and glycoside hydrolases (GH). In contrast, *Treponema* bins were associated with a higher abundance of carbohydrate binding modules (CBM), and tended to show higher prevalence of glycosyltransferases (GT), although not significantly (*P* = 0.10) (Fig. 5e).

10.1128/mSystems.00815-20.8 FIG S8 Phylogenetic similarity of reconstructed bins with reference genomes using single copy marker genes. (a) Phylogenetic trees showing the relationship of *Prevotella* genomic bins to reference *Prevotella* strains. (b) *Spirochaetaceae* genomic bins to reference *Treponema* strains. Maximum likelihood trees constructed using concatenated amino acid sequences from 30 (for *Prevotella*) and 29 (for *Treponema*) single copy marker loci, retrieved from *de novo* assemblies of shotgun metagenomic data. Recovered genomic bins were found similar to Prevotella copri DSM 18205 and Treponema succinifaciens DSM 2489, respectively. Download FIG S8, JPG file, 0.3 MB. Copyright © 2020 Sharma et al. 2020 Sharma et al. This content is distributed under the terms of the Creative Commons Attribution 4.0 International license.

To further break down these differences in broad CAZyme categories into more specific functions, relative abundances of significantly discriminating CAZy families were considered and plotted on a heatmap (Wilcox rank sum test, *P* < 0.05, Data Set S1, tab 17). Overall, most of the CAZy families enriched in *Prevotella* bins were involved in metabolism of cellulose/hemicellulose, xylan, pectin, xyloglucan, mannan, short-chain dextrins, fucose, interactions with cyclic oligosaccharides, and in the synthesis of glycoproteins (mannosyl-oligosaccharide alpha 1,2-alpha-mannosidase). In contrast, enzyme families enriched in *Treponema* bins were mainly involved in metabolic activities of glycogen, starch, peptidoglycan and in the metabolism of maltose (Fig. 5f and Data Set S1, tab 17). Then, the bins showing maximum completeness and closest similarity to the reference genomes were selected to assess their abundance distribution across all groups. Prevotella copri DSM 18205 (bin148) tended to show higher prevalence in BaAka hunter-gatherers and the gorillas during the dry season, although not significantly (Fig. S9a). In contrast, Treponema succinifaciens DSM 2489 (bin487) showed the greatest abundance in gorillas compared to both human groups, especially during the wet season; however, differences across seasons in gorillas were not significant (*P* = 0.3) (Fig. S9b).

10.1128/mSystems.00815-20.9 FIG S9 Abundance distribution of selected *Prevotella* and *Treponema* bins in the gut microbiome of BaAka hunter-gatherers, Bantu agriculturalists, and the gorillas during both seasons. (a) Relative abundance of *Prevotella* genome (bin148) and (b) relative abundance of *Treponema* genome (bin487). Download FIG S9, TIF file, 2.1 MB. Copyright © 2020 Sharma et al. 2020 Sharma et al. This content is distributed under the terms of the Creative Commons Attribution 4.0 International license.

## DISCUSSION

Research on the gut microbiome of traditional human populations and nonhuman primates has contributed significantly to our understanding of the ecological and evolutionary forces shaping the human microbiome (2, 6). Specifically, shifts in subsistence strategies and dietary choices are critical driving forces of the primate gut microbiome (7, 8, 20). These reports have emphasized the gain and loss of specific microbiome traits in primates, along with adaptations to energy-rich, processed diets, analogous to those characterizing agriculture and industrialization. In turn, rapid adaptations to these diets are believed to have had major impacts on human health (9, 11, 21–23). Here, we show that functional gut microbiome traits, which distinguish hunting and gathering from traditional agriculture in humans, parallel those seen in sympatric gorillas when shifting between diets of low and high energetic content. Building on this ecological gradient analogy, we sought to dissect the functional basis supporting microbiome similarities among different primate species and the ecological and dietary factors associated with loss and gain of specific microbiome features in humans.

These results show that exposure to agricultural and energy-dense diets in humans and nonhuman primates is characterized by an increased abundance of gut microbial transporters and transduction systems. Both functional traits appear to be enriched in the gut microbiome in response to energy-dense diets in mice and human models of obesity (24–28). Thus, high abundance of microbial transporters and transduction systems in gut microbiomes has been suggested to reflect an increased capacity to harvest dietary energy and exposure to a variety of dietary substrates, mainly diverse free sugars, but also lipids, peptides, metals, and even antibiotics (29, 30). Along these lines, we have previously shown that when gorillas shift from high structural polysaccharide intake associated with highly folivore diets to wet season-driven ripe fruit consumption (31, 32), their fecal metabolomes are substantially more diverse, reflecting wide exposure to different types of simple sugars, vitamins, lipids, amines, sterols, bile acids, indoles, and amino acids (13). Compared to hunter-gatherers, Bantu agriculturalists are also exposed to a wider range of dietary substrates from processed market foods, including greater proportions of energy-dense foods, rich in fat and free sugars and low in complex polysaccharides (12, 33).

Thus, exposure to diverse types of nutrients from energy-accessible diets may be a selective force causing gut microbiome similarities between traditional agriculturalists and the gorillas consuming ripe fruit. This dietary convergence may also be characterized by on-demand, individual access to a variety of foods, which may explain the high heterogeneity detected in gut microbial metabolic pathways and carbohydrate-active enzymes (CAZymes) in agriculturalists and gorillas during the wet season. We have previously shown increasing interindividual variability in microbiome taxonomic profiles in humans along subsistence gradients, from hunter-gatherers to traditional agriculturalists and humans in the United States (7). Indeed, when gorillas transition to energy-dense diets during wet seasons, when a variety of easily digestible foods is widely available (13), they exhibit marked interindividual differences in feeding behaviors within a single social group (31, 32, 34). Interindividual variation is also one of the main traits observed in the gut microbiome of humans on industrialized diets, which may be explained by significant heterogeneity in food choices (35). Such interindividual variability is believed to be associated with inconsistencies in individual responses to dietary or therapeutic interventions that target the microbiome to improve health, such as prebiotics (36, 37), antibiotics (38), and probiotics (39).

Dietary choices in hunter-gatherers are also substantially diverse (12, 40). However, in agricultural diets, dietary nutrients are likely consumed in free form and readily metabolizable by microbes and the host, as opposed to being embedded in natural, complex matrices with other nutrient fractions (i.e., fiber and phenolics) (21). This observation is concordant with more microbial adaptations for the degradation of broad plant polysaccharides, including lignified substrates, detected in the microbiomes of hunter-gatherers and gorillas during the dry season. However, carbohydrate-degrading capabilities involved in the metabolism of mannans, galactomannans, and glucomannans, which are widely distributed in legumes, seeds, nuts, tubers, gums, and fruit (41), were equally important in the BaAka and Bantu, and more prevalent in gorillas during the wet season. The functional overlap between BaAka and Bantu gut microbiomes may reflect shared dietary practices and the gradual integration of Congo basin hunter-gatherers into market economies and agriculture (12, 33). Nonetheless, the CAZyme repertoire of the BaAka was still significantly more similar to that of gorillas, regardless of season. The CAZyome overlap between the BaAka and gorillas indicates a stronger influence of complex polysaccharides in the BaAka diet and deviations from more natural microbiome configurations by the Bantu agriculturalists.

Also, compared with the BaAka or gorillas in the dry season, increased abundance of genes involved in the degradation of branched-chain amino acids (BCAAs) (leucine, valine, and isoleucine) was observed in the agriculturalists and in gorillas consuming ripe fruit. Increased colonic fermentation of BCAAs has been previously associated with a need for fermenting carbon sources other than carbohydrates, due to their low availability in the distal gut (42). A scenario of prolonged deprivation of carbohydrates accessible to microbes in the colon and increased BCAA metabolism and absorption has been associated with impaired insulin sensitivity and generation of toxic polyamines (43, 44). Along these lines, the prevalence of *Collinsella*, which characterized gorillas during the wet season and agriculturalists, in association with the abundance of transporters and transduction systems, has been also correlated with low dietary fiber intake, insulin resistance, and gut inflammation (45–47). Moreover, this taxon has been recently shown to influence the expression of host intestinal genes associated with certain metabolic syndromes (48). Thus, taxa such as *Collinsella* and an increased capacity to catabolize amino acids may constitute markers equally conserved in the human and nonhuman primate gut microbiome in response to low fiber availability in the colon.

Also, it is well established that carbohydrate and fat metabolisms are closely connected. Thus, it is expected that the downregulation of fat metabolism must occur in the face of increased carbohydrate consumption. This was evident in the negative associations found between genes involved in the synthesis and degradation of ketone bodies and both abundance and diversity of carbohydrate-active enzymes (Fig. S4c and d). Synthesis and metabolism of ketone bodies increase when there is limited carbohydrate colonic fermentation, in which case, microbes use ketone bodies as an alternative substrate for short-chain fatty acid (SCFA) generation (49, 50). The gut microbiomes of Bantu agriculturalists and gorillas during wet seasons may emphasize this metabolic route at the expense of colonic fiber fermentation.

Higher abundances of *Collinsella* also coincided with an increased capacity to degrade styrene in the Bantu agriculturalists. Styrene is an aromatic compound naturally found in plants, but it is also a toxic compound released into the environment by industrial chemical processes (51). The abundance of this xenobiotic degradation pathway in the Bantu agriculturalists is concordant with previously predicted adaptations to degrade bisphenol in this cohort and with reports of greater microbiome capacity to degrade xenobiotics in agriculturist and industrialized populations (7, 52, 53). The Bantu also exhibited the most unique XDE profiles compared to the hunter-gatherers and gorillas regardless of season; one example is the abundance of cytidine deaminase. This microbial enzyme mediates the catabolism of pyrimidine nucleoside xenobiotics, including the inactivation of therapeutic cancer drugs (54), which may reflect more access to commercial pharmaceuticals by traditional agriculturalists compared to foragers (33). Cytidine deaminase was also more prevalent in the wet season in gorillas. In this regard, the results of nutritional (14) and gut metabolomic analyses in western lowland gorillas (55) indicate higher exposure to a wide variety of compounds when the gorillas were consuming more fruit, including aromatic compounds (gallic, cinnamic, and coumaric acids), sterols, amines, indoles, and diverse nucleosides. Other enzymes involved in xenobiotic metabolism, such as methionine synthase and aconitate hydratase, which showed higher abundance in the hunter-gatherers and dry season in gorillas, are involved in folate and vitamin B_12_ biosynthesis and metabolism of short-chain fatty acids (56, 57), a characteristic of microbial ecosystems adapted to increased fiber degradation, as is the case in the bovine rumen (58). These observations confirm that increased dietary exposure to structural and other complex polysaccharides are important mediators of the convergence observed between traditional human populations and nonhuman primates. Nonetheless, the specific triggers of these analogous patterns in gorillas and traditional humans, in the context of dietary or other xenobiotic exposures, are unclear.

These data do not imply that all the microbiome features associated with traditional agriculture and consumption of ripe fruit by gorillas are proxies for the ecological basis of potentially detrimental diet-microbiome interactions. Taxa such as *Faecalibacterium* and Ruminococcus bromii, prevalent in agriculturalists and gorillas during the wet season, are not only known for their associations with consumption of resistant starch in cultivated plants, but also with immunomodulatory properties (59, 60). Likewise, Lactococcus lactis and *Leuconostoc* spp. have been linked to foodborne microbes associated with consumption of lacto-fermented foods, known for their immunomodulatory properties (61, 62). These microbiome markers may be reflective of technological and cultural innovations associated with traditional food processing and agriculture in humans (63, 64), while in gorillas, they may reflect dietary access to readily fermentable ripe fruits (32).

However, these data also emphasize gut microbiome markers that have been depleted in abundance along with the technological and cultural innovations associated with agriculture. Such is the case of *Prevotella* and taxa associated with the *Spirochaetaceae* family (e.g., *Treponema*), whose abundance in coprolites, nonhuman primates, and traditional populations and depletion in industrialized humans, has generated significant interest (17, 65–67). Here, it is shown that these taxa do not metabolize the same types of polysaccharides, and hence, their depletion in agriculturalists and wet season gorilla microbiomes are unlikely to be attributed to the same negative selective forces. These data support the contention that absence of *Prevotella* from the industrialized gut microbiome may be associated with loss of nutritionally diverse plant foods, composed of different types of complex and fermentable polysaccharides (11). This observation is concordant with the fact that *Prevotella* showed the highest diversity and richness of CAZymes and higher abundance of glycoside hydrolases and that this taxon is particularly enriched in primates with the most eclectic diets (5). In contrast, the loss of *Treponema*, whose abundance was higher in nonhuman primates compared to humans, regardless of subsistence strategy, may be associated with absence of very specific dietary substrates. Although genome reconstruction analyses of both taxa showed that they both exhibit significant glycogen- and starch-degrading capabilities, their specific dietary selective forces in traditional human populations still remain unclear and should be subject to further investigation.

### Study limitations.

One of the limitations of this study was not having controlled for physical activity and other lifestyle factors, which may also have an impact on the composition and function of the gut microbiome. Although it has been well documented that hunter-gatherers and gorillas during the wet season tend to have increased patterns of physical activity (22, 32, 68), it is unclear how this factor could affect microbiome function in the context of energy expenditure and metabolism in these populations. Likewise, even though dietary intake differences between the BaAka and Bantu, as well as gorillas during wet and dry seasons have been well established (32, 68), detailed diet intake data would have allowed us to investigate the associations between specific foods and the microbiome patterns observed. Last, we also acknowledge the limitations of our small sample size; however, studies with similar or greater sample sizes focusing on distinctions between the gut microbiome of hunter-gatherers, industrialized humans, and nonhuman primates across seasons of variable dietary intake have found similar patterns (8, 13, 52, 55).

### Conclusions.

In summary, these data emphasize parallel functional adaptations in the gut microbiome of humans and nonhuman primates in response to analogous ecological stimuli. Specifically, we highlight how adaptations to metabolize plant dietary polysaccharides, the degree of energy readily available in those foods, and degree of exposure to xenobiotics may be selective forces of conserved functional gut microbiome traits observed between nonhuman primates and traditional human populations. These parallel adaptations reflect loss of microbiome capabilities enabling the processing of a variety of complex polysaccharides, the emergence of taxa such as *Collinsella*, and very heterogeneous microbiome assortments among individuals, in association with traditional agriculture and energy-dense diets in gorillas. These traits, which have likely been exacerbated with industrialized lifestyles, have been associated with adverse metabolic phenotypes and inconsistent efficacy of specific dietary and therapeutic interventions targeted to improve metabolic health in industrialized populations through microbiome modulation. Although it cannot be stated that comparisons of contemporary primate populations are representative of evolutionary processes in human history, these data also shed light on the ecological processes associated with loss and gain of microbiome traits as humans have adapted to diverse dietary niches, including transitions to agriculture and industrialized, processed diets. Moving forward, it is critical to identify the specific dietary triggers of the microbiome traits conserved in traditional populations and nonhuman primates and to investigate whether their recovery in the guts of humans in culturally westernized contexts is conducive to signatures associated with improved metabolic health in subjects at risk.

## MATERIALS AND METHODS

### Sample collection.

Fecal samples of western lowland gorillas (*Gorilla gorilla gorilla*) were collected during November and December of 2009 (*n* = 11) (dry season), during June and July of 2011 (*n* = 12) (wet season). Fecal samples of BaAka hunter-gatherers (*n* = 14) and Bantu agriculturalists (*n* = 14) were collected during June to August of 2010 and 2011 at the Dzanga Sangha Protected Areas (DSPA), Central African Republic. Approximately 1 g of feces was placed in 2-ml Eppendorf tubes containing RNAlater (Invitrogen, Life Technologies). Samples were kept at room temperature for a maximum of one month before transport to the Institute of Vertebrate Biology, Czech Academy of Sciences, where they were kept frozen at −20°C, until they were shipped to the University of Illinois at Urbana-Champaign, where DNA was extracted. Ethical approval for sample collection and processing was granted by the Czech Academy of Sciences (55) and the University of Illinois Institutional Review Board for protection of human subjects (protocol number 13045, 4 September 2014) (7). The samples were collected noninvasively, adhering to DSPA research and ethical protocols and site regulations and approved by the Ministre de l’Education Nationale, de l’Alphabetisation, de l’Enseignement Superieur, et de la Recherche (Central African Republic).

### Dietary information of collected samples.

Western lowland gorilla fecal samples were collected in two seasons of different dietary intake. Each period differs substantially in terms of the availability of ripe fruits (14, 32, 34). During the dry season, gorillas heavily depend on a highly fibrous diet (mainly leaves, pith, bark, herbaceous vegetation, and other plant parts), due to the low availability of ripe fruit. In contrast, during the wet season, gorillas spend >80% consuming ripe, sweet fruit (31, 69, 70). Food items consumed in each season differ substantially in the content of phenolics and fibrous and readily digestible carbohydrates (32). The diets of BaAka hunter-gatherers mainly consist of wild and cultivated tubers and nuts, tree leaves (Gnetum africanum; high in fiber and tannins), honey, and wild game meat (7, 12, 71). Although the Bantu may overlap with the BaAka on consumption of some food items such as cultivated nuts and tubers, they rely more on agricultural products and a market economy, emphasizing higher consumption and intake frequency of grains, dairy products, and livestock (C. A. Jost Robinson, personal observation).

### Metagenomic sequencing and analysis.

DNA extraction from stool samples was conducted using the MoBio PowerSoil kit (then, MoBio Laboratories) and following the manufacturer’s instructions. Metagenomic libraries were constructed using the Kapa library preparation kit or the TruSeq SBS and sequenced on a HiSeq2500 Illumina sequencing platform at the Roy J. Carver Biotechnology Center, University of Illinois at Urbana-Champaign (gorilla samples) and at the J. Craig Venter Institute (La Jolla, CA), respectively. Then 2 × 150 paired-end (PE) reads were generated from an average 400 to 500 genomic DNA (gDNA) fragment size. The paired-end sequences were passed through FastQC for quality check (https://www.bioinformatics.babraham.ac.uk/projects/fastqc/), trimmed, and filtered to remove ambiguous bases (“N”) using the NGSQC toolkit (72). Homopolymer removal was conducted using prinseq (73), and reads were trimmed using a 10-bp sliding window with an average quality score of 20 and minimum length of 80 in Trimmomatic (74). These high-quality cleaned reads were filtered for gorilla DNA and human DNA via mapping them against gorilla genome (gorGor4, assembly date December 2014) and human genome (GRCh38, assembly date December 2013) using a combination of bowtie2, samtools, and bedtools (75–77). *De novo* metagenomic assembly was performed on these filtered sequences using metaSPAdes, with k-mer lengths of 21, 33, and 55 (78). Open reading frame (ORF) prediction was performed on the assembled contigs using prodigal (79). From the total number of genes, a nonredundant gene set (identity = 95%, alignment coverage = 90%) was created using CD-HIT (80) and used for gene quantification by aligning high-quality sequences using bwa and counting genes using samtools (81). Gene counts were further normalized by gene length and filtered for their presence in at least three samples. The relative abundances of each gene were used for downstream analysis.

KEGG pathway details, as far as carbohydrate- and xenobiotic-degrading abilities of these microbial communities were obtained using BLAST against the Kyoto Encyclopedia of Genes and Genomes database (KEGG_20032014), Carbohydrate-Active enzymes Database (CAZyDB_07202017), and xenobiotic-degrading enzymes (XDEs) downloaded from DrugBank, respectively, with a sequence identity threshold of 50%, query coverage fraction of 80%, a bit score of 60, and E value of 1e–6 (82–85). Taxonomic assignments were obtained from the alignment of all genes against NCBI and HMP databases (same version as used in our previous study (86) using comprehensive sequence similarity parameters across different phylogenetic ranks as described earlier (86, 87). The lowest common ancestor (LCA) method was used in case multiple best hits were found with equal identity percentage. Relative abundance of KO, pathways, CAZy families, XDEs, and microbial taxa were calculated using custom Perl scripts. These abundance tables were subsequently used for comparisons of functional and taxonomic potential between the four populations.

### Genome reconstruction.

Genome assembly was conducted to construct contigs using Megahit (88). High-quality reads were mapped on filtered contigs (>1000 bp) using bowtie2 (75) with default parameters, and the mean coverage of contigs was obtained using jgi_summarize_bam_contig_depths command of Metabat2 (89). Genomes were independently recovered from each population using Metabat2. Quality assessment of each genomic bin recovered was conducted using CheckM using lineage-specific marker genes and default parameters (90). The merge method of CheckM was used to combine bins from the same microbial population in order to increase the completeness (≥90) and reduce contamination (≤10). Additional bins were formed via grouping bins into a single bin if they met the defined criteria. For filtering contigs having divergent genomic properties, tetranucleotide frequencies were calculated using the outlier method of CheckM. Taxonomic string indicating approximate placement of the genomic bin in the tree was carried out using the tree_qa method of CheckM. Reference genomes of *Prevotella* and *Treponema* were downloaded from the NCBI and HMP databases. These reference genomes were used for inferring the evolutionary relationship of the specific genomic bins using ezTree (91), which identified single-copy marker genes from a group of genomes. Assembly statistics of selected bins were obtained using DFAST (92), and carbohydrate-degrading abilities were evaluated via BLAST alignment of protein-coding genes from these bins against the CAZyme database (84). The presence/absence matrix of CAZymes in selected bins was used for further statistical analysis.

### Statistical analyses.

Ordination analyses were performed using Bray-Curtis distances calculated on the relative abundance of KEGG pathways, CAZy families, XDEs, and bacterial species from metagenomic data, using the vegan and ape packages in R (93, 94). Differences between dry versus wet and BaAka versus Bantu were evaluated using the *adonis* function from the vegan package (95). Methods such as the two-sided Wilcoxon rank sum or Kruskal-Wallis test, species indicator analysis, and random forest were used for the identification of significantly discriminating taxa and functions, based on fold changes, *P* values, indval scores, and mean decrease in accuracy functions. Reporter feature algorithm was implemented to do gene set enrichment analysis on *P* values and fold changes of each KO for the identification of significantly discriminating pathways between different groups using the *runGSA* function in the R piano package (96). Heatmaps were generated using the *aheatmap* function from the NMF R package (97). The CCREPE R package was used to detect pairwise associations or coabundance patterns between selected bacterial species and functions (98). R packages dplyr and calibrate were used to format the data before plotting (99, 100). The relative abundances of CAZymes, calculated from presence/absence matrices, were used for principal-component analysis using the factoextra R package (101). All graphs and plots were generated using *ggplot*, *boxplot*, and Cytoscape 3.7.1 (102, 103).

### Availability of data and materials.

Shotgun metagenomic sequences generated in this study have been deposited in the NCBI SRA under the BioProject identifier (ID) code PRJNA635116.
