# Supplementary figures and images for: Traditional Human Populations and Nonhuman Primates Show Parallel Gut Microbiome Adaptations to Analogous Ecological Conditions
Source: mSystems. 2020 Dec 22;5(6):e00815-20. doi: 10.1128/mSystems.00815-20 (PMC7762792; doi:10.1128/mSystems.00815-20)

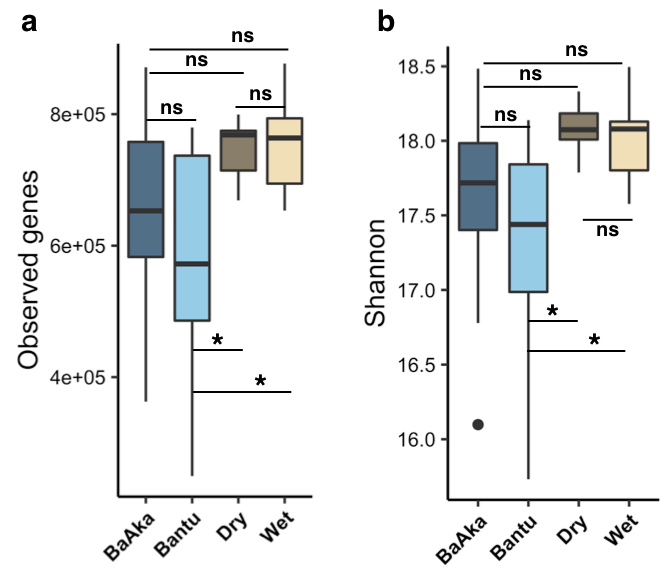

Supplement: FIG S1 [file mSystems.00815-20-sf001.tif]

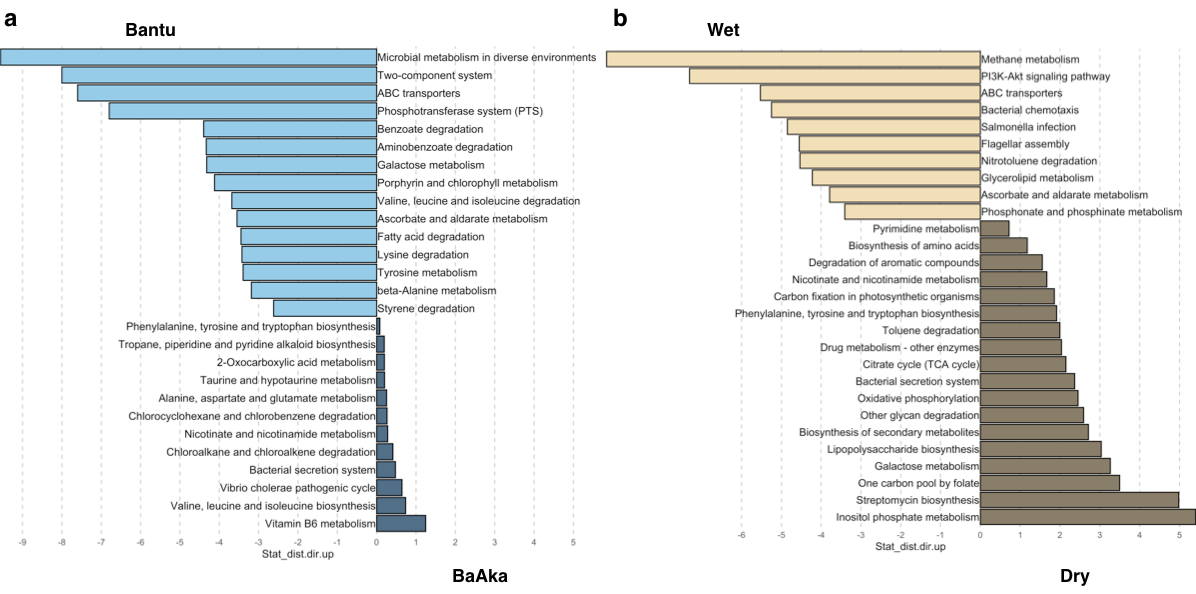

Supplement: FIG S2 [file mSystems.00815-20-sf002.tif]

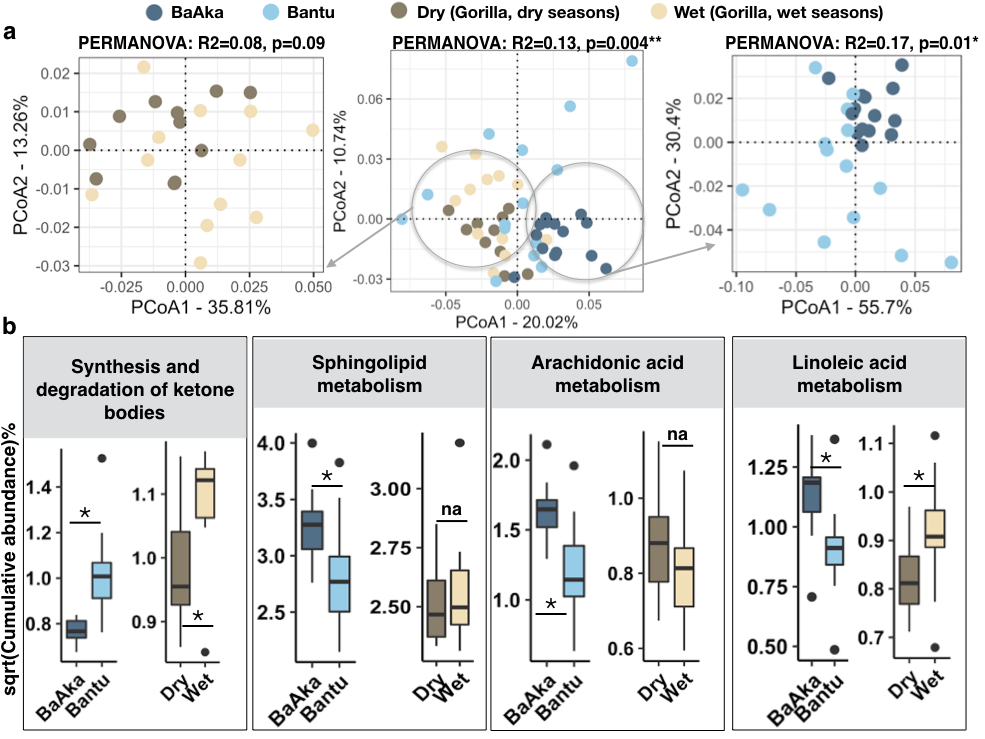

Supplement: FIG S3 [file mSystems.00815-20-sf003.tif]

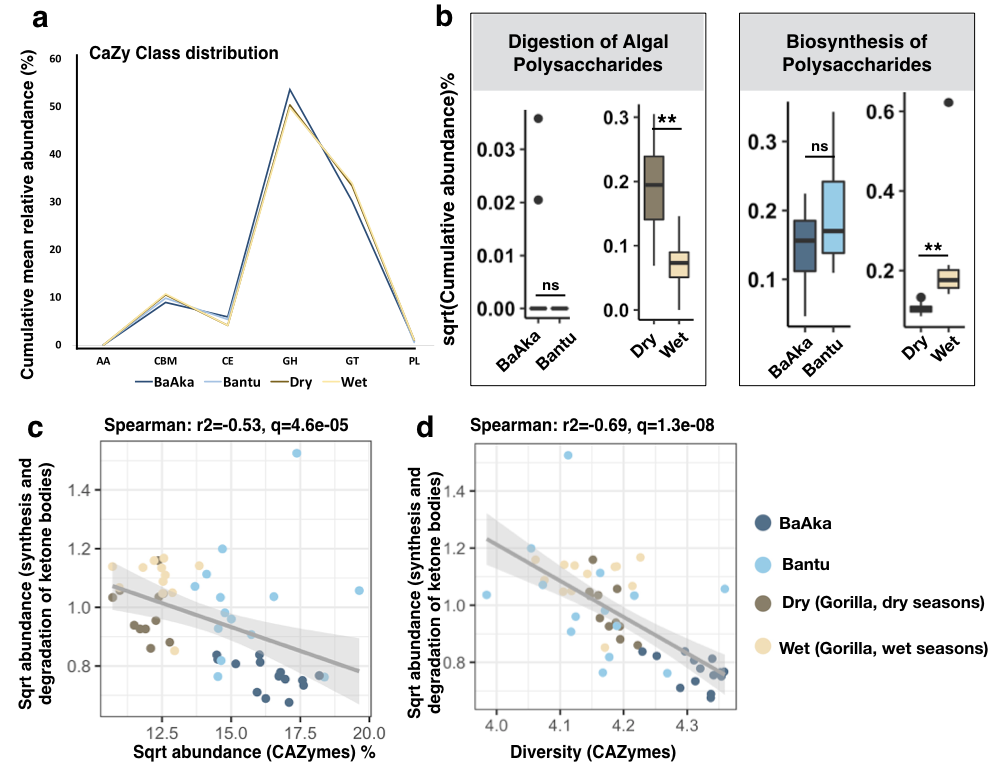

Supplement: FIG S4 [file mSystems.00815-20-sf004.tif]

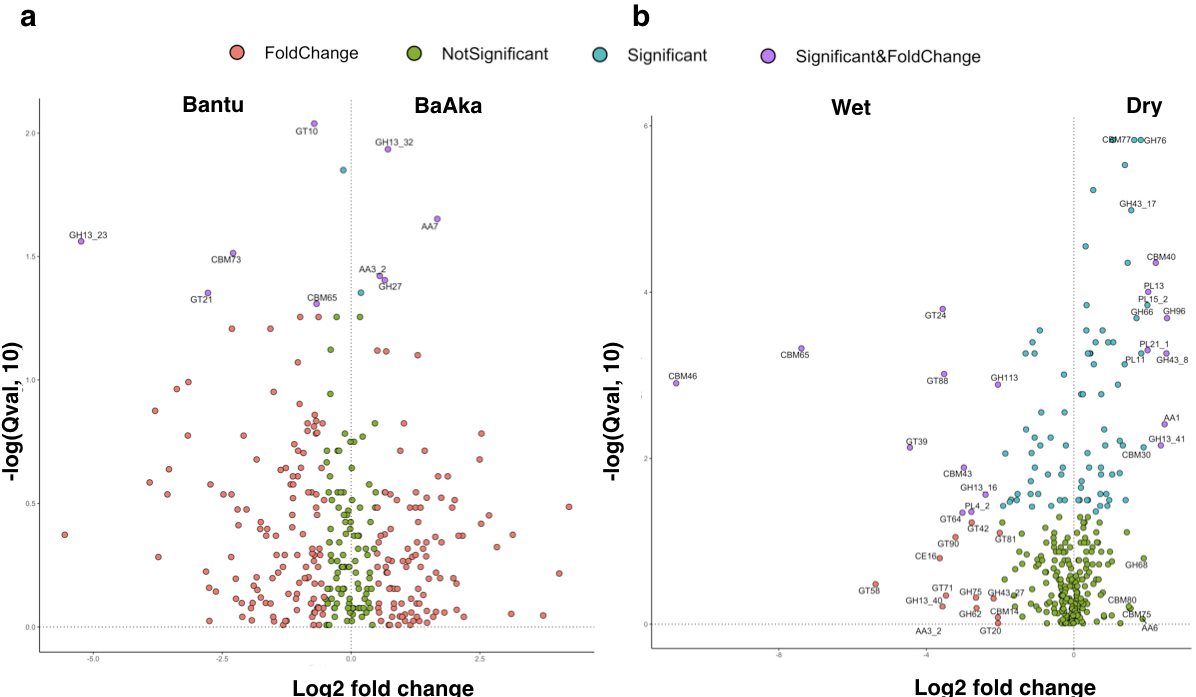

Supplement: FIG S5 [file mSystems.00815-20-sf005.jpg]

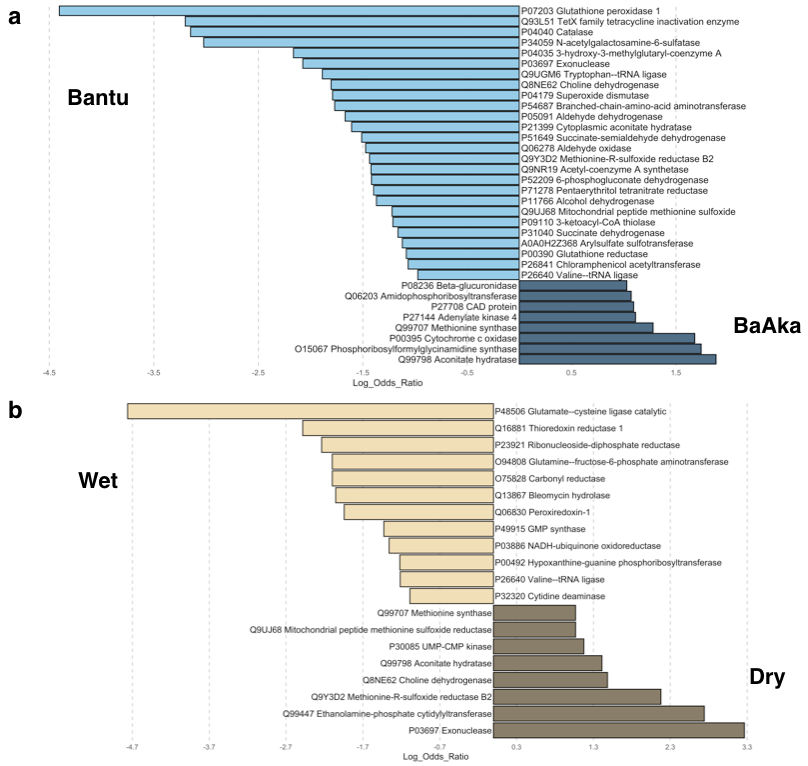

Supplement: FIG S6 [file mSystems.00815-20-sf006.tif]

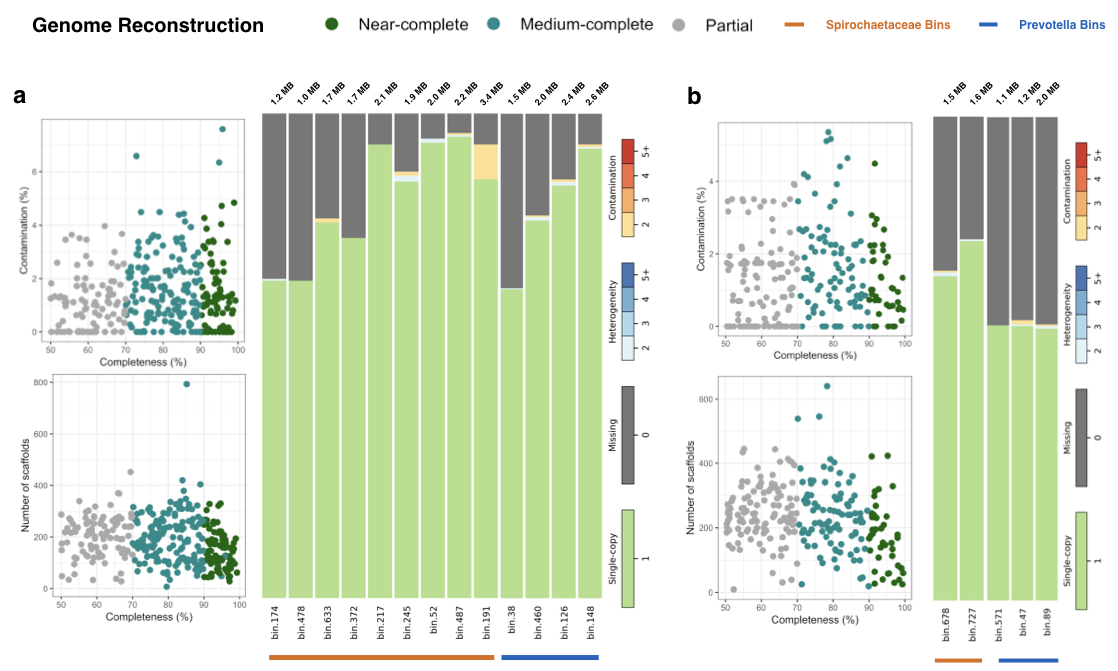

Supplement: FIG S7 [file mSystems.00815-20-sf007.tif]

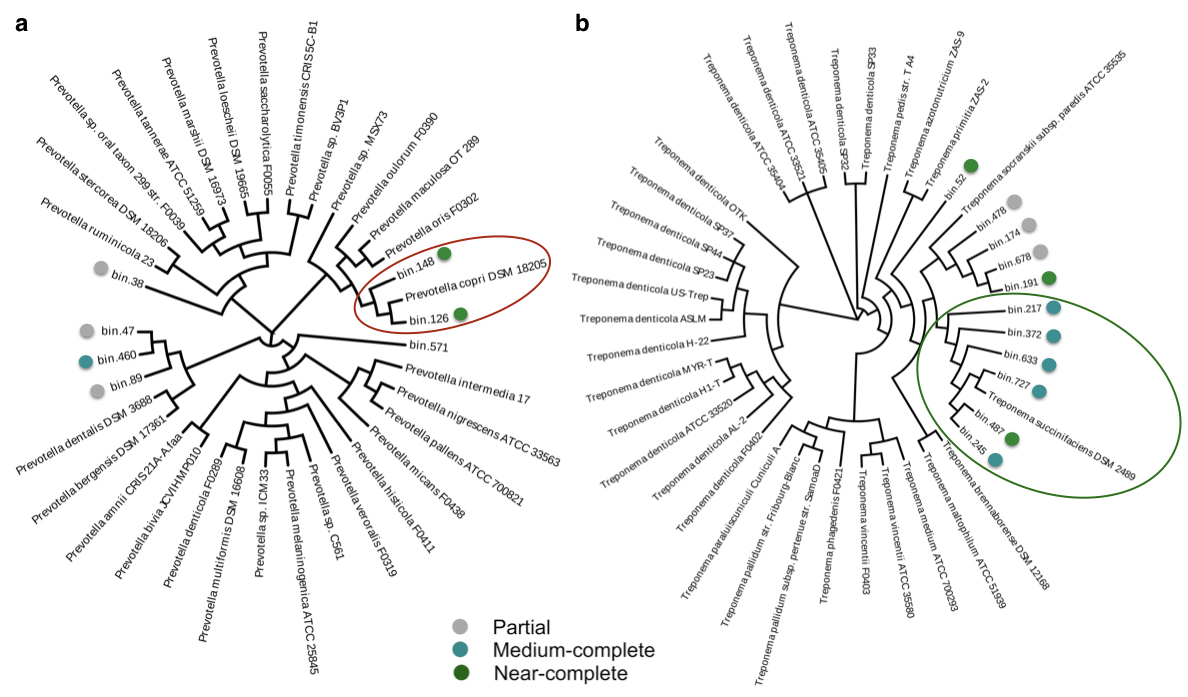

Supplement: FIG S8 [file mSystems.00815-20-sf008.jpg]

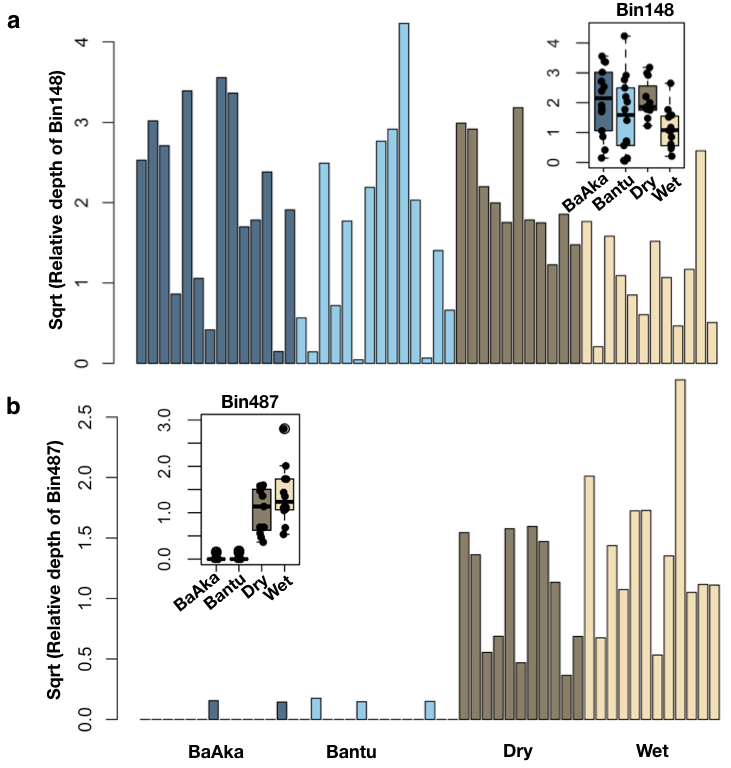

Supplement: FIG S9 [file mSystems.00815-20-sf009.tif]
